# Comparative Genomics of Bacteriophage of the Genus *Seuratvirus*

**DOI:** 10.1093/gbe/evx275

**Published:** 2017-12-20

**Authors:** Pavelas Sazinas, Tamsin Redgwell, Branko Rihtman, Aurelija Grigonyte, Slawomir Michniewski, David J Scanlan, Jon Hobman, Andrew Millard

**Affiliations:** 1 Department of Biotechnology and Biomedicine, Technical University of Denmark, Lyngby, Denmark; 2 School of Life Sciences, University of Warwick, Coventry, United Kingdom; 3 Warwick Medical School, University of Warwick, Coventry, United Kingdom; 4 School of Biosciences, University of Nottingham, Sutton Bonington Campus, Sutton Bonington, United Kingdom; 5 Department of Infection, Immunity and Inflammation, University of Leicester, United Kingdom

**Keywords:** bacteriophage, genomics, evolution

## Abstract

Despite being more abundant and having smaller genomes than their bacterial host, relatively few bacteriophages have had their genomes sequenced. Here, we isolated 14 bacteriophages from cattle slurry and performed de novo genome sequencing, assembly, and annotation. The commonly used marker genes *polB* and *terL* showed these bacteriophages to be closely related to members of the genus *Seuratvirus*. We performed a core-gene analysis using the 14 new and four closely related genomes. A total of 58 core genes were identified, the majority of which has no known function. These genes were used to construct a core-gene phylogeny, the results of which confirmed the new isolates to be part of the genus *Seuratvirus* and expanded the number of species within this genus to four. All bacteriophages within the genus contained the genes *queCDE* encoding enzymes involved in queuosine biosynthesis. We suggest these genes are carried as a mechanism to modify DNA in order to protect these bacteriophages against host endonucleases.

## Introduction

Viruses are thought to be the most abundant biological entities on the planet, with an estimated 10^31^ present in the biosphere (Suttle 2007). However, the number of genomes of viruses specifically infecting bacteria (bacteriophages) lags well behind those of their hosts (Sepulveda et al. 2016; Casey et al. 2017; Hatfull 2015). Thus, there are currently ∼400 genomes of bacteriophages within the European Nucleotide Archive (ENA) that infect “Enterobacteria” or *Escherichia*, compared with the ∼66,000 *Escherichia* genomes that are publicly available (https://enterobase.warwick.ac.uk/; last accessed December 28, 2017). The diversity of bacteriophages is exemplified by the number of novel genes found within their genomes with many bacteriophages having little similarity at the genomic level (Pope et al. 2015). In this study, we isolated and sequenced 14 bacteriophages from a single source of cow slurry gathered from a dairy farm slurry tank in Leicestershire, to expand the diversity and number of bacteriophages that infect *Escherichia coli*.

## Materials and Methods

Bacteriophages were isolated from a single sample of cattle slurry that was collected from a farm in the United Kingdom in 2016. Bacteriophages were isolated using the double-agar overlay method, and purified through two rounds of double-agar overlay (Kropinski et al. 2009). All bacteriophages consistently produced clear plaques and thus are thought to be lytic.

DNA was extracted from 1 ml of bacteriophage lysate as previously described (Rihtman et al. 2016). Sequencing was performed on an Illumina MiSeq (250 bp paired-end), utilizing 1 ng of input DNA for Nextera XT library preparation, following the manufacturer’s instructions. Prior to assembly, reads were trimmed with Sickle using default parameters (Joshi and Fass 2011). Assembly was carried out with SPAdes v.3.6.0 using assembler only options (Bankevich et al. 2012). The resulting genomes were annotated with Prokka 1.12 using a custom database of proteins extracted from all current viral genomes [April 2017] as well as using pVOGS (Seemann 2014; Grazziotin et al. 2017). Single gene phylogenetic analysis was carried out using TranslatorX to build nucleotide alignments using MAFFT, based on the aligned amino acid sequence (Katoh and Standley 2013). Phylogenetic trees were constructed using FastTree v2.1.4 (Price et al. 2010). Comparative genome analysis was carried out using Roary, with “-I 50” (Page et al. 2015). A core-gene phylogeny was constructed using FastTree v2.1.4 (Price et al. 2010). Core-gene data were visualized with Phandango (Hadfield et al. 2018). ANI was calculated using autoANI.pl with default settings (Davis II et al. 2016). All genome sequences were submitted to the ENA under the project accession PRJEB22133.

## Results

### Genome Features

**Fig. 1.**
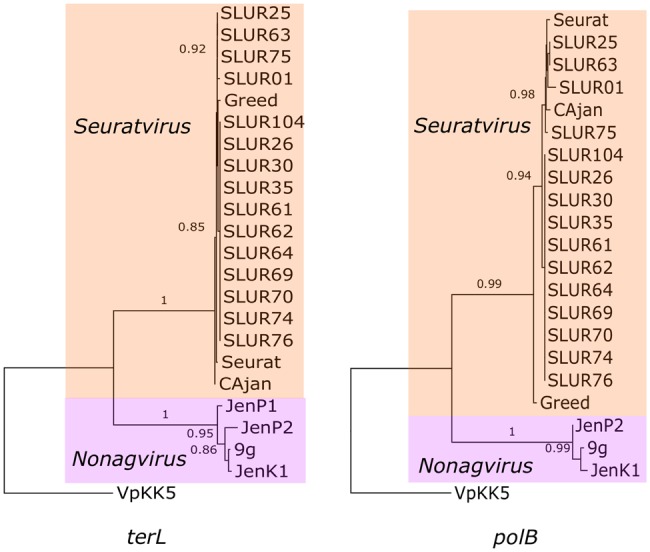
—A phylogenetic tree of bacteriophage *polB* and *terL* sequences from the genus *Seuratvirus*. Nucleotide alignments were built on the translated nucleotide sequence. A generalized time-reversible model of evolution was used. Bacteriophages; SLUR25, SLUR63, SLUR75, SLUR01 (LN881725.1), SLUR104, SLUR26, Greed (KX534337), SLUR26, SLUR30, SLUR35, SLUR61, SLUR62, SLUR64, SLUR69, SLUR70, SLUR74, SLUR76, Seurat (KM236243.1), CAjan (KP064094.1), JenP1 (KP719132.1), JenP2(KP719133.1), 9g (KJ419279.1), Jenk1(KP719134.1). Bacteriophage VpKK5 was included as an outgroup (KM378617).

The genomes of the 14 newly isolated bacteriophages were assembled into complete chromosomes that ranged in size from 58.998 kb to 60.165 kb, with a G + C content that varied from 44.43% to 44.79%. The genomes were predicted to have between 87 and 92 genes (median 91), with no tRNAs detected. We used the gene encoding a large subunit terminase (*terL*) and DNA polymerase subunit B (*polB*) to determine the phylogeny of these bacteriophages. Both analyses indicated that the isolates from this study are distantly related to bacteriophages of the genus *Nonagvirus*, but form a monophyletic clade with bacteriophages of the genus *Seuratvirus* and are part of the *Siphoviridae* family (fig. 1).

## Comparative Genomics

**Fig. 2.**
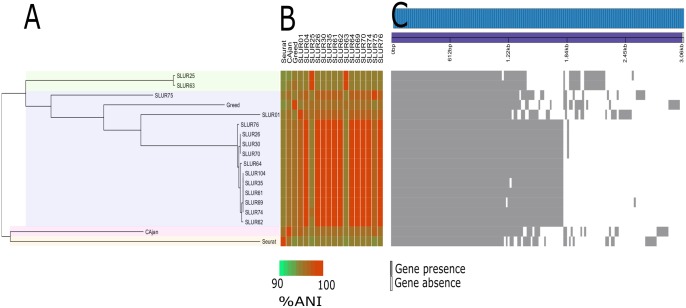
—Diversity of bacteriophages in the genus *Seuratvirus*. (*A*) Phylogeny based on 58 concatenated core-genes. (*B*) Average nucleotide identity. (*C*) Gene presence–absence.

To further understand the evolution of the newly isolated bacteriophages they were compared at the genome level to all other bacteriophages using the BlastN algorithm. Only four previously sequenced bacteriophages shared >20% nucleotide identity over >75% of their genome. Two of these bacteriophages Seurat (Doan et al. 2015) and CAjan (Carstens et al. 2016) are part of the genus *Seuratvirus* providing further evidence that the new isolates are part of this genus, with the remaining bacteriophages SLUR01 (Smith et al. 2015) and Greed (Malki et al. 2016) so far unclassified by the International Committee on Taxonomy of Viruses (ICTV). The genomes of CAjan, Greed, SLUR01, and Seurat were included in a comparative analysis with the 14 new bacteriophage isolates. All bacteriophage were found to share a similar genome architecture (supplementary figs. 1 and 2, Supplementary Material online). The core-genome of the 18 bacteriophages was determined using Roary (Page et al. 2015), with a set of 58 core genes identified, accounting for approximately two-thirds of the genome. To infer evolutionary relationships between the phage isolates, the 58 core genes were used for further phylogenetic analysis (fig. 2). Bacteriophage CAjan and Seurat both formed distinct groups, supporting their previous designation as two different species. Additionally, a further two distinct clades were observed, with the largest containing 11 new bacteriophage isolates (fig. 2). Therefore, two novel putative species within the genus *Seuratvirus* were identified.

To further compare all 18 bacteriophages, the average nucleotide identity (ANI) of each bacteriophage was computed and clustered using an all-against-all approach (see supplementary fig. 3, Supplementary Material online). The resulting clusters of bacteriophages are congruent with the core-gene analysis. Current guidelines for defining a phage species suggest that as a starting point any two bacteriophages with an ANI > 95% should be considered as the same species (Adriaenssens and Brister 2017). The two named species of Seurat and CAjan have an ANI of ∼96%; the addition of a further 16 bacteriophages results in all 18 bacteriophages having an ANI of >95% with at least one other bacteriophage isolate (fig. 2, supplementary fig. 3, Supplementary Material online). Simply using this 95% ANI cut-off would result in collapsing this group of bacteriophages into one single species, therefore obscuring the diversity observed with the core-gene phylogeny (fig. 2) and the marker genes *polB* and *terL* (fig. 1). Increasing the ANI threshold to 97% for this group of bacteriophages would result in four species represented by the bacteriophages Seurat, CAjan, SLUR63/25, and all other remaining bacteriophages (fig. 2). While this is still not in complete agreement with the core-gene analysis, it is nearly congruent, with the only exception being bacteriophage Greed. Using ANI alone, bacteriophage Greed would be classified as a single species, though this is not supported by the core-gene analysis.

**Fig. 3.**
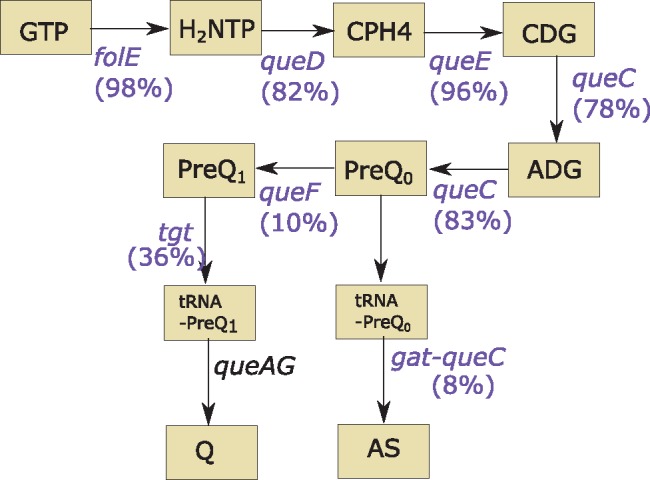
—The biosynthetic pathway for the synthesis of 7-deazaguanine derivatives (Thiaville et al. 2016). A total of 60 bacteriophages were found to contain genes related to the biosynthesis of 7-deazaguanine derivatives. The frequency at which each gene occurred in these bacteriophage genomes is marked in brackets. Substrates are: ADG, 7-amido-7-deazaguanine; CDG, 5-carboxydeazaguanine; CPH4, 6-carboxytetrahydropterin; AS, archeosine; GCHI, GTP cyclohydrolase I (FolE); H2NTP, dihydroneopterinphosphate; TGT, tRNA-guanine transglycosylase; Q, queosine. Genes that are found in bacteriophage genomes are colored purple.

A noticeable feature of all the bacteriophages isolated in this study was the presence of genes encoding proteins involved in queuosine biosynthesis. Queuosine is a hyper-modified guanosine analogue, first discovered at position 34 of the anticodon loop of tRNA^Try^ in *E. coli* (Harada and Nishimura 1972). The distribution of modified nucleosides in tRNAs and pathways for their insertion have both been studied. The pathway for modification of guanosine in tRNAs has been found within both prokaryotes and eukaryotes (Vinayak and Pathak 2009). In bacteria, modification of tRNAs requires four essential genes (*queC*, *queD*, *queE*, and *queF*; Reader et al. 2004). Genes involved in the biosynthesis of queuosine have also previously been identified in bacteriophages and viral metagenomes (Smith et al. 2015; Kulikov et al. 2014; Holmfeldt et al. 2013). Using the pVOG database (Grazziotin et al. 2017), we systematically searched for genes related to queuosine biosynthesis in currently available bacteriophage genomes using the following VOGs: VOG5156 (*queD*), VOG1020 (*queC*), VOG0998 (*queE*) VOG6942 (*queF*), and VOG322 (GTP cyclohydrolase I, *folE*). We identified 60 bacteriophages containing one or more queuosine-related genes (supplementary table 1, Supplementary Material online). These genes were only found within members of the *Myoviridae* and *Siphoviridae* families. Only six viruses had all four known essential genes for queuosine biosynthesis. These viruses infect a diverse range of hosts, including *Haloarcula*, *Cellulophaga*, and *Streptococcus* and no do not fall within a single viral taxonomic group (supplementary table 1, Supplementary Material online). Whereas only six bacteriophages contained all four essential genes, the majority of bacteriophages did contain three of the four essential genes with *queF* being the least frequently found (fig. 3). Consistent with other bacteriophages all the newly isolated bacteriophages contained *queCDE*, as well as a gene encoding GTP cyclohydrolase I. Because all the known members of the genus *Seuratvirus* contain homologues of *queCDE*, the presence of these genes can be considered a distinguishing feature of the genus.

## Discussion

The isolation of 14 new bacteriophages specifically capable of infecting *E. coli*, has further expanded the diversity of known bacteriophages. Before 2015 there were no cultured representatives of the genus *Seuratvirus*. Here, we show they can be readily isolated from the environment. In addition, the new isolates further expand the genus *Seuratvirus*, and through the use of core-gene phylogeny combined with ANI we suggest the genus currently contains four different species. Furthermore, for this group of bacteriophages using an ANI cut-off of >95% is not appropriate to approximate the diversity observed within core-gene analysis. An ANI of >97% would better resemble the observed phylogeny and should be used for delineating bacteriophage within this genus.

We had previously isolated bacteriophage SLUR01 in 2015 from the same slurry tank as the new isolates (in 2016). The isolation of further bacteriophages from the same species, suggests that there is a stable population of closely related bacteriophages within this system over a 12 month period. Furthermore, this demonstrates that genetically similar bacteriophages can be isolated from geographically distant places, for example, UK (SLUR bacteriophages), Denmark (CAjan), and USA (Seurat, Greed) as well as from a range of environments. Despite their geographic and environmental differences these isolates have genomes that are remarkably similar in terms of both gene content and synteny.

The presence of queuosine genes is a signature found in all genomes of the genus *Seuratvirus*. The conserved order of these genes and the presence of the same genes colocalized in other bacteriophages suggests they may be part of a module that is transferred amongst bacteriophages, although the lack of sequence similarity at the nucleotide level suggests some divergence. The function of the queuosine biosynthesis genes remains unknown. Queuosine is a well-known nucleoside derivative that modifies tRNAs by replacement of guanine at position 34. However, the biological role of queuosine-modification in bacteria is still unclear (Vinayak and Pathak 2009), with some evidence that is improves reading frame maintenance (Urbonavičius et al. 2001), but a recent study demonstrating *E. coli* cells lacking modified tRNAs have no significant difference in growth rate compared with an isogenic wild type control (Xu et al. 2016). If these genes do serve to modify tRNAs in the same way in bacteriophages as they do in their host, the benefit to the bacteriophage is not unclear. Alternatively, these genes may act to modify bacteriophage DNA and function as a protection mechanism against restriction endonucleases, as has recently been suggested (Kulikov et al. 2014; Thiaville et al. 2016). It has also been shown that 7-deazaguanine derivatives, with queuosine one such derivative, are inserted into the DNA of *Salmonella* (Thiaville et al. 2016). Furthermore, bacteriophage 9g, which is part of the genus *Nonagvirus* has homologues of queuosine biosynthesis genes and is known to both modify its DNA, converting deoxyguanosine to 2′-dexoy-archeosine (Thiaville et al. 2016), and be resistant to a wide range of restriction endonucleases (Kulikov et al. 2014). Thus, modification of bacteriophage DNA is thought to provide a protection mechanism against host endonucleases during infection. The queuosine biosynthesis related genes found within the genus *Seuratvirus* are unlikely to have exactly the same function as observed for bacteriophage 9g as they lack homologues of *gat-queC* which is required for the insertion of archeosine (Thiaville et al. 2016; fig. 3). Our analysis showed that *gat-queC* homologues are found only in bacteriophages of the genus *Nonagvirus*, distinguishing them from all other bacteriophage genera that contain *queC* homologues. Bacteriophage of the genus *Seuratvirus* contain only a *queC* homologue and therefore likely modify their DNA with different derivatives of 7-deazaguanosine to that of the *Nonagvirus*. We propose that bacteriophage of the genus *Seuratvirus* modify their DNA to provide a protection mechanism against host endonucleases in a similar manner to bacteriophage 9g (Kulikov et al. 2014), but that this modification is more likely to be the insertion of a queuosine rather than archeosine. Further experimental work is required to determine if this is the case and whether bacteriophage carries these genes to supplement or replace host copies of these genes. The absence of *queF*, an essential gene in queuosine biosynthesis, suggests they may be supplementing a host biosynthetic function, possibly relieving a metabolic bottleneck. Alternatively, these phage genes may replace the host metabolic genes completely because one of the many phage-encoded hypothetical genes potentially acts in an analogous manner to QueF, to produce PreQ_1_, the final intermediate in the production of queuosine (fig. 3).

## Supplementary Material

Supplementary data are available at *Genome Biology and Evolution* online.

## Supplementary Material

Supplementary Data
