# Supplementary material for: Comparative Genomics of Bacteriophage of the Genus Seuratvirus
Source: Genome Biol Evol. 2017 Dec 20;10(1):72–6. doi: 10.1093/gbe/evx275 (PMC5758909; doi:10.1093/gbe/evx275)
Supplement: Supplementary Data [file evx275_supp.zip › Supplementary Figure Legends.docx]

Supplementary Figure 1

Comparison of all bacteriophage genomes to the type bacteriophage Seurat. BRIGG was used for genome comparison using the blastn algorithm. The inner circle shows the GC skew for the genome of bacteriophage Seurat, with genes marked with black arrows, core-genes are shaded in grey. The outer circles represent each bacteriophage isolate, with the shading of each ring representative of the nucleotide identity (from 80-100 %, as determined by BLAST), the dark the shading the higher identity.

Supplementary Figure 2

Genome map of bacteriophage SLUR25. All bacteriophage shared a similar genome architecture with the genome architecture represented by SLUR25. Genes are represented by arrows. Genes that are coloured in red have a putative function assigned to them, with genes shaded in purple have no known function. Selected genes with a putative function have been labelled.

Supplementary Figure 3

In an all-v-all approach, bacteriophages were compared to determine average nucleotide identity. Clustering of bacteriophage isolates was based on Average nucleotide identity. Bacteriophages were clustered on Euclidean distance using average linkage.

Supplementary Table 1 - Distribution of queuosine biosynthesis related genes in bacteriophage genomes
